# Biological control agent *Rhizobium* (=*Agrobacterium*) *vitis* strain ARK-1 suppresses expression of the essential and non-essential *vir* genes of tumorigenic *R. vitis*

**DOI:** 10.1186/s13104-018-4038-6

**Published:** 2019-01-03

**Authors:** Akira Kawaguchi, Mizuho Nita, Tomoya Ishii, Megumi Watanabe, Yoshiteru Noutoshi

**Affiliations:** 1 Western Region Agricultural Research Center, National Agriculture and Food Research Organization (NARO), 6-12-1 Nishifukatsu-cho, Fukuyama, Hiroshima 721-8514 Japan; 2 AHS Jr. Agricultural Research and Extension Center, School of Plant and Environmental Sciences, Virginia Polytechnic Institute and State University, 595 Laurel Grove Rd, Winchester, VA 22602 USA; 3 Graduate School of Environmental and Life Science, Okayama University, 1-1-1 Tsushima-naka, Kita-ku, Okayama, 700-8530 Japan

**Keywords:** *Rhizobium vitis*, Grapevine crown gall, Biological control, Gene expression, Acetosyringone

## Abstract

**Objective:**

To gain insights into the virulence suppressive mechanism of a nonpathogenic strain of *Rhizobium vitis* ARK-1, we co-inoculated ARK-1 with a tumorigenic (Ti) strain of *R. vitis* to examine the expression of two essential virulence genes (*virA* and *virG*) and one non-essential gene (*virD3*) of the Ti strain at the wound site of grapevine.

**Results:**

Co-inoculation of ARK-1 with a Ti strain VAT03-9 at a 1:1 cell ratio into grapevine shoots resulted in significantly lower expression of the virulence genes *virA, virD3,* and *virG* of VAT03-9 at 1 day after inoculation compared with those when shoots were inoculated only with VAT03-9. ARK-1 was not able to catabolize acetosyringone, which is the plant-derived metabolites inducing the entire *vir* regulon in Ti strains, suggesting the direct effect of ARK-1 on the induction of broad range of *vir* genes of *R. vitis* Ti strains.

**Electronic supplementary material:**

The online version of this article (10.1186/s13104-018-4038-6) contains supplementary material, which is available to authorized users.

## Introduction

Grapevine (*Vitis vinifera* L.) crown gall is caused mainly by *Rhizobium vitis* (Ti) [syn. *Agrobacterium vitis* (Ti)], where “Ti” means “tumorigenic”. To avoid confusion, we follow the nomenclature for *Rhizobium* species adopted by Young et al. [1]. Crown gall is one of the most important diseases of grapevine around the world [2, 3]. Infected vines generally lose their productivity, and rapid decline can be associated with the infection of young vines.

The virulence (*vir*) genes and transfer DNA (T-DNA) are located mostly in large tumor-inducing plasmids (pTi). *Rhizobium* Ti strains transfer T-DNA and several virulence effector proteins into plant host cells, and this infection pathway is mediated by a bacterial type IV secretion system [4, 5]. The plant phenolics acetosyringone (AS) and α-hydroxyacetosyringone induce the entire *vir* regulon in *Rhizobium* as well as the formation of T-DNA intermediate molecules [4]. T-DNA transfer and processing require products of the several *vir* genes, which are named as *virA* to *virE*, and *virG* and located outside of the T-DNA coding region [4–7].

Previously, we have reported that a nonpathogenic *R. vitis* strain VAR03-1, which was isolated from grapevine in Japan and strongly inhibited tumor formation in tomato, grapevine, rose, sunflower, and apple [8–11]. Moreover, we isolated and identified nonpathogenic *R. vitis* strain ARK-1, which performed much better than VAR03-1 at inhibiting tumor formation in grapevine in greenhouse and field trials, as a new antagonistic strain [12–16]. ARK-1 is also endophytic in grapevine [12]. When grapevine shoots were inoculated with a Ti strain that was not affected by ARK-1 in the antibiosis assay, ARK-1 was able to suppress tumor formation. [13]. In addition, dead cells of ARK-1 (autoclaved) and the culture filtrate (CF) of ARK-1 (without cells) were not able to inhibit tumor formation in grapevine [15]. When ARK-1 and a Ti strain was co-inoculated, the number of colony-forming unit (cfu) of the Ti strain was not affected from 1 to 5 days after inoculation (dai), but it was significantly reduced at 7 dai [13, 14].

Saito et al. [17] have reported that the suppressive activity of antagonistic and non-pathogenic *R. vitis* strain VAR03-1 on the virulence gene expression of Ti was found to be its CF in vitro. Consistent with our speculation, the cfu of Ti strain was temporarily reduced after incubation of CF prepared from the growth medium of VAR03-1. Interestingly, the suppressive activity was detected in the high molecular weight fraction (> 100 kDa) of CF, suggesting that the antagonistic effects of VAR03-1 on Ti are mediated by large particles released in the culture media [17]. On the other hand, the CF of ARK-1 did not show suppressive effect on both the tumor formation and the expression of *vir* genes *in planta* experiments [14].

Two different mechanisms (antibiotic compounds or quorum-sensing) of biological control of plant crown gall disease using antagonistic bacteria have been reported [8–10, 17–23], but disease suppression mechanism of ARK-1 is different from these two mechanisms [13, 14, 16]. The biological control activity of ARK-1 is likely based on the suppression of some essential virulence genes [14, 16]. Two *Rhizobium* proteins, VirD2 and VirE2 expressed by *virD* and *virE*, respectively, are directly associated with the T-strand [4–6]. Co-inoculation of grapevine shoots with ARK-1 and Ti strain at a 1:1 cell ratio resulted in significantly lower expression of the virulence genes *virD2* and *virE2* of Ti strain at 1 dai than expression levels of these genes by a Ti strain inoculated by itself [14, 16]. When a non-pathogenic *R. vitis* strain VAR06-30, which is neither antagonistic against *R. vitis* (Ti) nor limit the development of crown gall of grapevine, was co-inoculated with a Ti strain, expression levels of *virD2* and *virE2* were not affected (Additional file 1: Table S1), [14].

At this moment, it remains unclear if ARK-1 suppresses the expression of the other *vir* genes including essential or non-essential genes. Two *Rhizobium* proteins, VirA and VirG are directly associated with the T-strand as essential *vir* genes. VirA molecule works as the sensor protein to recognize the plant signal molecule AS. VirG protein works as the response regulator, which activates all genes in the regulon [4–6]. On the other hands, there are some non-essential genes such as *virD3* in *vir* regulon, which are not required for tumorigenicity on plants [24].

To gain insights into the virulence suppressive mechanism of ARK-1, we co-inoculated ARK-1 with a Ti strain to examine the expression of two essential virulence genes (*virA* and *virG*) and one non-essential gene (*virD3*) of the Ti strain at the wound site of grapevine.

## Main text

### Methods

#### Detection of *vir* genes’ mRNA using the RT-qPCR

Cell suspensions of the non-pathogenic strains ARK-1 (antagonistic and non-pathogenic), VAR06-30 (non-antagonistic and non-pathogenic), and VAT03-9 (tumorigenic) (Additional file 1: Table S1) were prepared from 48-h-old cultures on potato sucrose agar medium slants [12, 14, 15]. Supernatant of these cultures was discarded, then the surface of the slant was washed with distilled water to obtain a cell suspension. Cell concentration was adjusted to approximately 10^8^ cells mL^−1^. Two mixed cell suspensions at a cell ratio of 1:1 (ARK-1 plus VAT03-9, VAR06-30 plus VAT03-9), and a VAT03-9 suspension were prepared. An inoculation method was followed the needle-prick method using grapevine seedlings grown from a seed (2 years old, cv. ‘Neo Muscat’, seeds obtained from grape clusters cultivated in NARO, Japan) [14]. Each seedling (one plant per pot) was inoculated once with one of the mixed cell suspensions or with only VAT03-9. Seedlings inoculated with sterile distilled water were used as the negative control. We grew the seedlings in a greenhouse at 20 to 35 °C with natural sunlight and collected the shoot samples that included the one wound site. One sample, which was 0.2 g fresh weight, was obtained per plant from five plants (i.e., *n* = 5) at 1 dai.

The basic information for the RT-qPCR procedures, which followed the methods of Bustin et al. [25] and Kawaguchi [14], are summarized in Table 1 and the Additional file 2: Table S2. A housekeeping gene *pyrG* of Ti strain VAT03-9 was amplified to be used as both for the standardization of amplified products and an internal control [14]. To amplify the *pyrG* gene of VAT03-9 alone and to not amplify the same gene of strains ARK-1 and/or VAR06-30, specific primers and probe were designed and confirmed that these primers/probes worked well [14].

**Table 1:** Oligonucleotide primers and probes used in the RT-qPCR analyses

Name	Primer or probe	5′-3′ nucleotide sequence	Target DNA	Reference	Accession no.	Design or supplier
virA-F	Primer	CTC GAC TGA AAG AAG GAC ATA CTC A	*virA*	This study	CP000637	Licensed Dual-Labeled Probes for qPCR (Sigma-Aldrich)
virA-R	Primer	CGC AAG AAG GTC TAT CAT AAG GAA G	*virA*	This study	CP000637	Licensed Dual-Labeled Probes for qPCR (Sigma-Aldrich)
virA-P	Probe	AAT GGT TCC GCT CGC AAG CAC CTT G	*virA*	This study	CP000637	Licensed Dual-Labeled Probes for qPCR (Sigma-Aldrich)
virD3-F1P	Primer	GTC AAC TCG GGG AAG TCG T	*virD3*	This study	CP000637	ProbeFinder (Roche Diagnostics)
virD3-R1P	Primer	CAG TTC AGT TCG ATC GCT TG	*virD3*	This study	CP000637	ProbeFinder (Roche Diagnostics)
virD3-P	Probe	TTC CTC TG	*virD3*	This study	CP000637	Universal ProbeLibrary probe #6 Cat. no. 04685032001 (Roche Diagnostics)
virG-F	Primer	CTG TCT CGG ATG CGA GTA CG	*virG*	This study	CP000637	Licensed Dual-Labeled Probes for qPCR (Sigma-Aldrich)
virG-R	Primer	CGG AGT ATC TAA CGA TCC ACG C	*virG*	This study	CP000637	Licensed Dual-Labeled Probes for qPCR (Sigma-Aldrich)
virG-P	Probe	TGA CCG CCG TAT CCG ATA GTC AGC AAT T	*virG*	This study	CP000637	Licensed Dual-Labeled Probes for qPCR (Sigma-Aldrich)
VAT03-9pyrG-F-2	Primer	GGT GAA GTC TTC GTC ACC GAC	*pyrG*	[14]	AB272142	Licensed Dual-Labeled Probes for qPCR (Sigma-Aldrich)
VAT03-9pyrG-R-2	Primer	CGC CCT GTG AAG CGT TCA TAG	*pyrG*	[14]	AB272142	Licensed Dual-Labeled Probes for qPCR (Sigma-Aldrich)
VAT03-9pyrG-P-2	Probe	CGG CGC AGA GAC CGA CCT TGA TCT TG	*pyrG*	[14]	AB272142	Licensed Dual-Labeled Probes for qPCR (Sigma-Aldrich)

Relative quantification of the *virA*, *virD3*, and *virG* genes’ mRNA concentrations was carried out using the ΔΔCt method [26] by the Ct values of *pyrG* gene’s mRNA as reference points [14] across three independent technical replications. Relative expression rate of each *vir* gene in grapevine inoculated with ARK-1 plus VAT03-9 and VAR06-30 plus VAT03-9 was reported as percentage of the expression of each gene in grapevine inoculated with only VAT03-9 strain. All measurements were taken at 1 dai. The means of five biological replicates were used as a measured relative expression level.

The effect of treatment, gene, and their interaction on the median adjusted gene expression rate was examined using the linear mixed model ANOVA [packages ‘car’ and ‘lme4’ in R, ver. 3.5 (http://www.r-project.org/)]. In the model, treatment, gene, and their interaction were considered as fixed effects, and experimental replication was considered as a random effect. For the effect of treatment, Tukey’s HSD (package ‘emmeans’ in R) was used as a post hoc multiple comparison method.

#### Catabolism test of AS

Cell suspensions (2 mL each) of ARK-1, VAR06-30, and VAT03-9 were prepared from 24-h-old cultures on King’s B medium [27] in a shaking incubator at 28 °C. The tubes with each culture were centrifuged at 4000 rpm for 15 min. A supernatant of these strains was discarded and the surface was washed with distilled water to obtain a cell suspension, which was suspended in AT minimal media solution [28–30] with 0.2% mannitol as a carbon source (called ATM) with supplemental biotin (2 μg mL^−1^). The cell concentration was adjusted to approximately 2 × 10^8^ cells mL^−1^. Two millilitre of cell suspensions were prepared in three tubes per strain. Three tubes containing 2 mL of ATM were also prepared as a negative control. The 2 μL of AS (Sigma-Aldrich, Germany) was dissolved in dimethyl sulfoxide (DMSO) to produce a 10 mM stock solution. The 2 μL of the stock AS solution was applied to each tube of ARK-1, VAR06-30, VAT03-9, or the negative control. Each tube was incubated in a shaking incubator at 28 °C for 24-h. Then, 1 mL of culture supernatant was obtained from each culture after centrifugation with 13,000 rpm for 5 min. Then, concentration of AS in each culture fluid was measured at UV 296 nm after 1.95 min using the high-performance liquid chromatography (HPLC, HITACHI LaChrom Elite, Japan) equipped with Kinetex 2.6 μm Biphenyl column (150 × 4.6 mm) (Phenomenex, USA). The experiment was repeated three times. The effect of treatment on the concentration of AS was examined with a linear mixed model ANOVA as described above.

### Results

#### Suppressive effect on expression of the *vir* genes of the Ti strain by co-inoculation with ARK-1 in grapevine plants

**Fig. 1:**
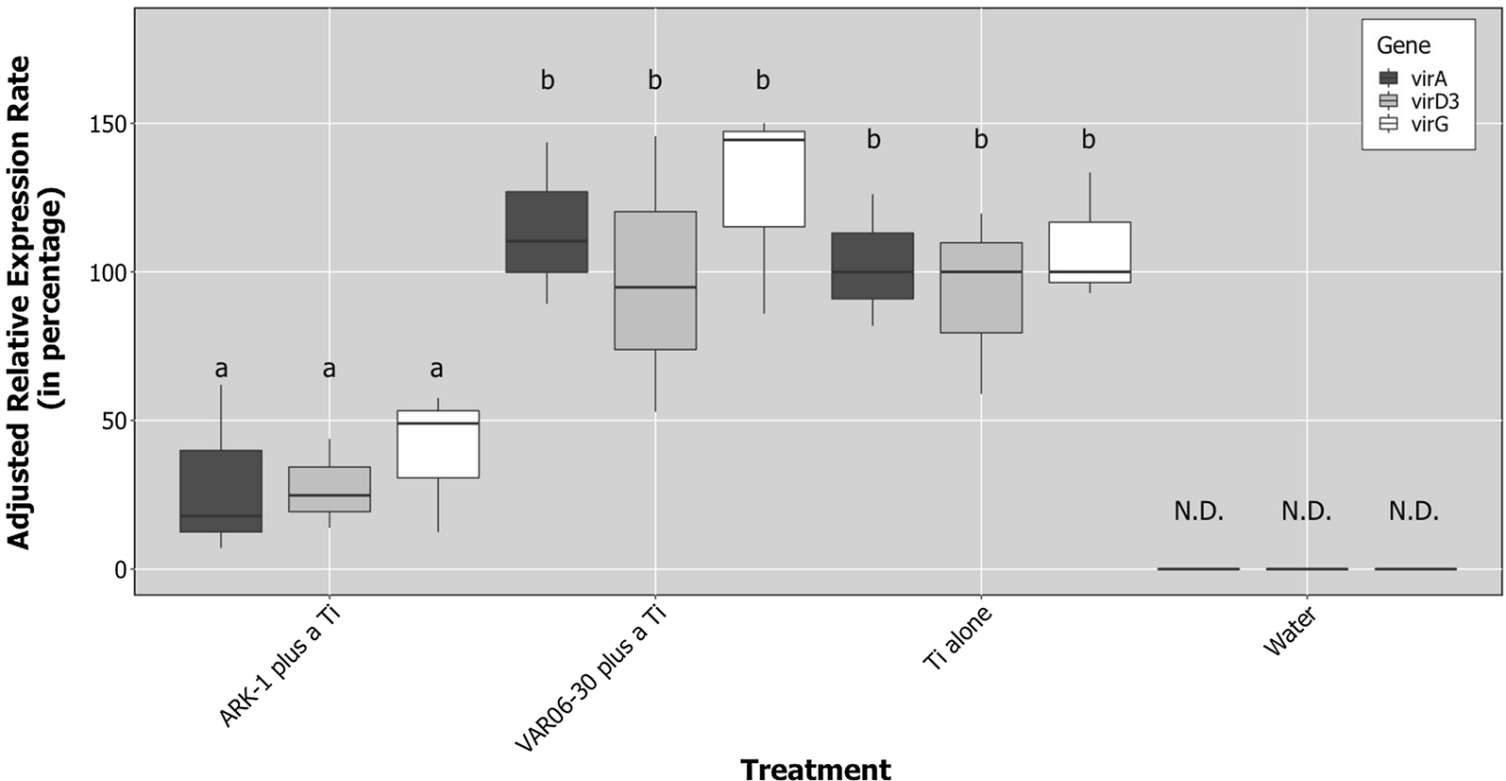
Relative expression rate of the *virA*, *virD3,* and *virG* genes in bacterial cells of the *Rhizobium vitis* Ti strain in grapevine plants measured using the reverse transcriptase quantitative polymerase chain reaction (RT-qPCR). Values (%) represent the relative expression rate of the *vir* genes at 1 day after inoculation (dai) compared with the value when only Ti strain VAT03-9 was inoculated (“Ti strain alone”), which had a value of 100%. Each cell suspension or mixture (ARK-1 plus a Ti, VAR06-30 plus a Ti, and a Ti alone) at cell ratios of 1:1 was inoculated onto the stems of grapevine plants after wounding. Stem samples were harvested at 1 dai. The center bar of the boxplot is the median, the lower and upper horizontal bars are 25th and 75th percentile, and whiskers show 95% range. Boxes labeled with different letters indicate a significant difference from the other bars in same gene expression (*P* ≤ 0.05, Tukey’s HSD test)

The treatment factor significantly affected the median adjusted expression rate (*F* = 21.9, *P* < 0.01), but the effect of gene (*F* = 1.0, *P* = 0.38) and treatment and gene interaction (*F *= 0.09, *P* = 0.98) were not significant. The median adjusted expression rate of *virA*, *virD3*, and *virG* genes were significantly suppressed (*P* ≤ 0.05) by co-inoculation of ARK-1 with VAT03-9 (Fig. 1). On the other hand, no significant difference (*P* > 0.05) in the adjusted median expression rate of *virA*, *virD3*, and *virG* were observed when co-inoculation of VAR06-30 with VAT03-9 and inoculation of VAT03-9 alone were compared (Fig. 1).

#### Catabolization of AS by ARK-1

**Fig. 2:**
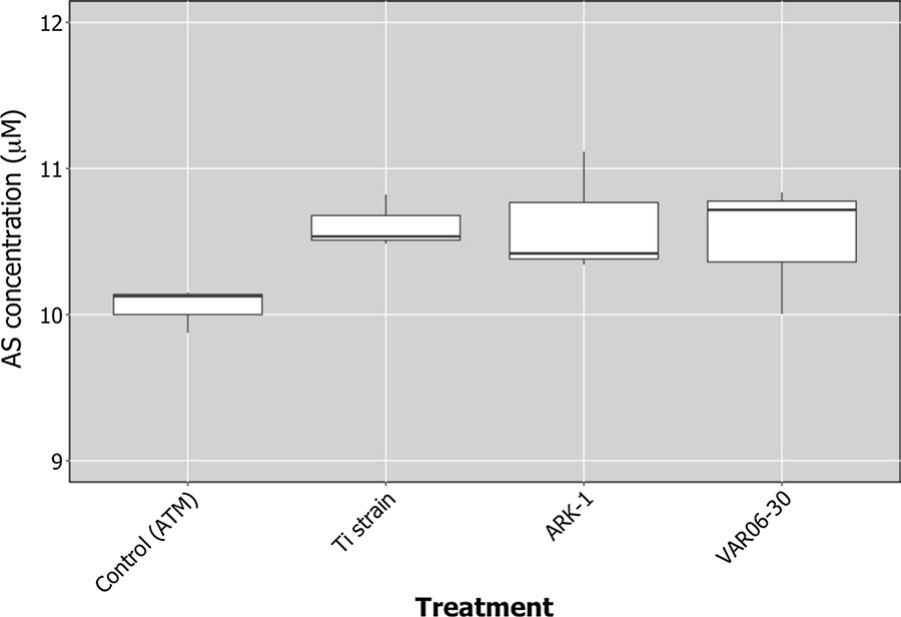
The concentration of acetosyringone (AS) after incubation *Rhizobium vitis* Ti strain (VAT03-9) and non-pathogenic strains (ARK-1 and VAR06-30) with AT medium (ATM) or only ATM without a bacterial strain as negative control. The center bar of the boxplot is the median, the lower and upper horizontal bars are 25th and 75th percentile, and whiskers show 95% range

There were no significant differences in the concentrations of AS among each *R. vitis* strain (*F* = 2.2, *P* = 0.19). No significant reduction of AS content was observed compared with the negative control (Fig. 2).

### Discussions

In our study, treatment with ARK-1 suppressed the expression of *virA*, *virD3*, and *virG* by the Ti strain in grapevine. In comparison, VAR06-30, a non-antagonistic and non-pathogenic strain, did not suppress the expression of these genes. With results from this and a previous study [14], ARK-1 has shown to be able to suppress the expression of at least five different *vir* genes: *virA*; *virD3*; *virG* (this study); *virD2*; and *virE2* [14]. ARK-1 suppresses both essential (*virA*, *virD2*, *virE2,* and *virG*) and non-essential (*virD3*) virulence genes of *R. vitis* Ti strains. Since ARK-1 suppresses the expression of *virA* and *virG*, which are the first two triggers of expression of all other *vir* genes in *Rhizobium* Ti strains [4–6], there is a possibility that all subsequent expression of *vir* genes can be suppressed.

We previously demonstrated that ARK-1 suppressed the expression of *vir* genes of Ti when the expression was triggered by AS in a liquid culture medium [14]. However, there was a possibility that the suppression of these *vir* genes was a consequence of quick catabolization of AS by ARK-1, thus, the sensor protein VirA was not able to detect AS to trigger subsequent *vir* gene expressions [4–6].

In catabolization test, however, all the tested *R. vitis* strains including ARK-1was not able to catabolize AS (Fig. 2). This result indicates that ARK-1 is not capable of metabolizing AS to interfere with a Ti strain’s *vir* genes. Therefore, ARK-1 has an ability to suppress the expression of *vir* genes via other mechanism(s).

## Conclusions

ARK-1 suppressed expression of the virulence genes *virA*, *virD3*, and *virG* of a Ti strain at the wound site without catabolizing AS. The suppressive effect of ARK-1 was not specific to the essential virulence genes.

## Limitations

We have carried out the inoculation with grapevines seedlings, not adult trees.

## Additional files

**Additional file 1: Table S1.** Bacterial strains used in this study.

**Additional file 2: Table S2.** Experimental condition used in reverse-transcriptase quantitative real-time polymerase chain reaction (RT-qPCR) based on MIQE requirements.

## Abbreviations

CF: culture filtrate
AS: acetosyringone
RT-qPCR: reverse transcriptase quantitative polymerase chain reaction
DMSO: dimethyl sulfoxide
